# A novel stacking ensemble model for predicting discharge coefficient of submerged multi parallel radial gates

**DOI:** 10.1038/s41598-026-38117-2

**Published:** 2026-03-03

**Authors:** Noran M. Abdelazim, Mohamed Hosny, Fahmy S. Abdelhaleem, Ahmed M. Elshenhab, Amir Ibrahim

**Affiliations:** 1 Department of Civil Engineering, Canadian International College, New Cairo, 1059 Egypt; 2 Center for Finance and Digital Economy, King Fahd University of Petroleum & Minerals, 31261 Dhahran, Saudi Arabia; 3 Department of Civil Engineering, Benha Faculty of Engineering, Benha University, Benha, 13512 Egypt; 4 Department of Mathematics, Faculty of Science, Mansoura University, Mansoura, 35516 Egypt

**Keywords:** Radial gates, Submerged flow, Ensemble learning, Long short-term memory, Machine learning, Discharge coefficient, Engineering, Mathematics and computing

## Abstract

Enhancing the precision of discharge coefficient (C_d_) prediction holds paramount importance for effective Water distribution control. Calculating the C_d_ for radial gates is often complex, with existing methods frequently depending on intricate procedures and underlying assumptions. This study introduces a deep learning-based stacking ensemble model for C_d_ prediction. The proposed model comprises a dual-layer structure. Four machine learning algorithms are exploited as baseline models. The Meta model employed long short-term memory (LSTM) with attention mechanism to amalgamate the outputs from the base models and assign sufficient weight to each base model. The spatial attention mechanism effectively highlighted relevant patterns within the data. The proposed model achieved an impressive root mean square error of 0.0175. The ensemble model outperformed existing longstanding models. The proposed system holds substantial strategic importance, enabling optimal water resource management.

**Supplementary Information:**

The online version contains supplementary material available at 10.1038/s41598-026-38117-2.

## Introduction

Flow regulator structures hold pivotal significance in the equitable distribution of flow within irrigation systems. The efficacy of these structures stands as a linchpin determining the success of irrigation systems. Although the design of such systems predominantly hinges on the maximum discharge criterion, real-world scenarios seldom align with this assumption. Consequently, in such systems, the deployment of flow regulator structures becomes imperative. These structures serve a dual purpose: facilitating gravity-based irrigation conditions and ensuring precise water level adjustments to meet specific requirements^1^. Commonly, irrigation projects prominently feature radial gates. Reviewing the literature revealed that, many researches were found on radial gates^2–7^. Upon which project operators heavily rely on precise discharge measurements, a fundamental element in delivering water effectively to end-users, flow characterization remains an ongoing topic of investigation, with many researchers contributing to this field^8–10^. These radial gates eliminate the need for separate structures dedicated solely to flow measurement^5^. However, a notable challenge in flow measurement arises from the calibration of these radial gates, primarily due to structural nuances, the diversity of gate types and the susceptibility of calibrations to factors like the seal type and downstream channel width^11^. Calibration procedures for gates operating under free flow conditions are well-documented in standard references, known for their ease of use and high accuracy. In contrast, gates functioning under submerged flow conditions often face calibration inaccuracies, with error rates reaching up to 50%^12^. Extensive global research on the hydraulic performance of gates has greatly enhanced understanding of their applications. Radial gates are the most commonly used type, mainly because they can withstand the substantial forces required for operation, a feature that hydraulic engineers often prefer over other gate designs. This preference is largely due to the unique design of radial gates, which feature cylindrical shells that allow water pressure to pass through the gate axis without creating torque forces^13^. Therefore, accurate calculation of C_d_ for radial gates holds significant importance. Bijankhan et al.^14^ delved into a comprehensive analysis of radial gate performance under different flow conditions, employing dimensional analysis and Buckingham theorem to introduce a novel dimensional equation for C_d_. Meanwhile, Salmasi et al.^13^ explored the impact of various sill shapes beneath radial gates. Onward, they assessed two distinct sill placements, revealing that sills could exert C_d_ positively and negatively.

In parallel, Zheng et al.^15^ developed a model based on the least square method for discharge calibration in radial gates, ultimately enhancing accuracy across all methods and significantly reducing the relative error to a mere 3.54%. However, it’s worth noting that these C_d_ equations rely on assumptions that may yield inflated results. Besides, empirical C_d_ formulas encompass elevated uncertainty, a multitude of influential parameters, intricate interactions, numerous assumptions and solution complexity. In response to these challenges, machine learning (ML) as a promising tool in hydraulic engineering is on the rise^16–19^ and has exhibited notable success in modelling C_d_^20–22^. ML models can effectively handle equations subject to boundary conditions, providing a reliable means to determine C_d_. ML models can offer a direct avenue for addressing issues of conventional methods because of their capacity to resolve difficult nonlinear situations^23^. Authors in^24^ harnessed genetic programming to ascertain C_d_ for inclined slide gates. While in^25^, a neuro-fuzzy model is leveraged to predict C_d_ for labyrinth side weirs, employing 285 experimental datasets to validate its effectiveness. In^18^, oblique sluice gate C_d_ was examined using various techniques, with the artificial neural network (ANN) approach emerging as the most accurate model. Roushangar et al.^26^ introduced an extreme learning machine algorithm for C_d_ prediction in submerged radial gates. Their research demonstrated the model robustness in estimating C_d_ under varying submergence conditions. The upstream Froude number Fr and the ratio of downstream water depth to gate opening y3/e were found to be key factors influencing Cd. Cao et al.^27^ used Random Forest (RF) and its optimization to build an intelligent prediction model that analyzes the hydraulic characteristics of sluice gate submerged flow. Cd falls with y3/e and rises with Fr. In parallel, Rady^28^ applied ANN to explore C_d_ for vertical and inclined sluice gates operating under two flow conditions. His findings underscored the remarkable capabilities of ANN in modelling flow rates, achieving accuracy levels within $\:\pm\:$5%. Existing literature on C_d_ determination for radial gates often involves solving complex nonlinear equations and analysing detailed graphs. While many studies have explored empirical formulas, these formulas are typically limited by specific datasets and channel characteristics, making them less applicable to a broader range of channels and flow types.

Machine learning (ML) models have emerged as promising solutions due to their ease of application and high precision. However, previous research has highlighted several limitations^29,30^. Many studies used small experimental datasets, which limits the model’s applicability in real-world scenarios. Additionally, these models often require extensive internal parameter optimization^12,31^ and complex model architectures^32^. Furthermore, significant developer intervention is needed to address challenges like overfitting and data dependency. The reliance on experimental data and limited sample sizes restricts the models generalizability across diverse environments. In recent years, machine learning (ML) and deep learning (DL) techniques have been increasingly adopted in a wide range of hydrological and hydraulic forecasting applications beyond discharge coefficient estimation. These applications include rainfall–runoff modeling, streamflow and runoff forecasting, sediment transport prediction, groundwater level estimation, and flow regime classification^33,34^. Advanced DL architectures such as long short-term memory (LSTM), convolutional neural networks (CNN), and attention-based models have demonstrated strong capabilities in capturing nonlinear dependencies, temporal dynamics, and complex interactions among hydrological variables [11535 36, 37]. Furthermore, hybrid and ensemble ML–DL frameworks have gained considerable attention due to their ability to combine complementary strengths of different learners, improve robustness, and reduce prediction uncertainty^38,39^. Recent studies have shown that stacking and hybrid ensemble strategies can outperform standalone models in flow-related forecasting problems by dynamically integrating information from multiple base predictors. These developments highlight the growing potential of ensemble learning and DL-based meta-models as powerful tools for solving complex hydraulic engineering problems, motivating their application to discharge coefficient prediction in submerged radial gate systems.

Ensemble learning models combine multiple learners to reduce prediction variance, mitigate overfitting, and enhance overall performance^40^. ANN, Gaussian process regression (GPR) and support vector machines (SVM) are widely used for C_d_ predictions, ensemble learning algorithms like stacking have been largely unexplored. Additionally, although deep learning (DL) techniques have been extensively applied to estimate C_d_ in various hydraulic structures such as weirs, their application in predicting C_d_ for radial gates remains relatively limited. This study aims to overcome existing limitations by utilizing the stacking ensemble algorithm for C_d_ prediction. We introduce a novel ensemble strategy integrated with DL technology, enhancing both accuracy and real-world applicability. Unlike previous studies that relied on experimental data, the presented approach uses field data, which significantly improves the system’s relevance and effectiveness in practical scenarios. The proposed model is built upon Long Short-Term Memory (LSTM) model with an attention mechanism as the meta model, for the purpose of predicting C_d_ in radial gates. In this way, GPR^1,12,41^, SVM^32,42^, ANN^18,28,42,44^, and Least-squares boosting (LSBoost)^45^ were adopted as the base models within the stacking approach. The selection of these models is strategic, as they represent prominent techniques in C_d_ prediction. Furthermore, these four models exhibit complementary strengths and weaknesses, enhancing the effectiveness of the developed stacking model. This approach enables us to explore how deep learning (DL) can analyze the relationships between input parameters and C_d_ to develop a reliable C_d_ calculation system. To assess the performance of the proposed model, we compared its results with those from linear and non-linear regression models, including the Generalized Linear Model (GLM), Generalized Regression Neural Network (GRNN), Regression Neural Network (RNN) and bagging. This comparative analysis is crucial for ensuring reliable results and evaluating our model against established methods for C_d_ estimation.

Despite the growing use of machine learning techniques for discharge coefficient (Cd) prediction in hydraulic structures, existing studies have predominantly focused on single standalone models and simple ensemble strategies, with limited attention to advanced deep learning–based stacking frameworks, particularly for submerged multi-parallel radial gates. In this context, the present study introduces a novel deep learning-based stacking ensemble architecture that advances the state of the art in hydraulic gate discharge modelling. The proposed approach is distinguished by three key contributions. First, it represents the first application of a stacking ensemble framework tailored specifically for predicting the discharge coefficient of submerged multi-parallel radial gates, addressing a gap in current hydraulic engineering literature. Second, an attention-based long short-term memory (LSTM) network is employed as a meta-learner, enabling adaptive and data-driven weighting of heterogeneous base machine learning models, rather than relying on fixed or heuristic aggregation schemes commonly adopted in previous studies. This design allows the ensemble to dynamically emphasize the most informative predictors under varying hydraulic conditions. Third, unlike many earlier investigations that rely primarily on limited laboratory experiments, the proposed framework is developed and validated using a large-scale field dataset collected from operational irrigation regulators, thereby enhancing the model’s robustness, generalizability, and practical relevance for real-world water management applications. Collectively, these contributions not only improve prediction accuracy but also provide a scalable and operationally meaningful framework for integrating advanced deep learning techniques into hydraulic gate calibration and decision-support systems.

## Materials and methods

### Dataset description

This study focuses on three regulators situated within the Delta Irrigation District, Delta Barrages, Egypt: Al-Tawfiki, Al-Menoufi, and Abasi regulators. These structures are designed for automatic operation under submerged flow conditions. Al-Tawfiki regulator, positioned 965 km downstream of the Aswan old dam (AOD) and upstream of the Damietta Barrage, boasts a capacity of up to 20 million cubic meters per day (MCM/d) and benefiting 1.60 million acres of farmland. It comprises six radial gates, each spanning 5 m in width and separated by five piers, each 2 m wide. Meanwhile, Al-Menoufi regulator, also located 965 km downstream of AOD and upstream of Damietta Barrage, possesses a capacity of up to 25 MCM/d, benefiting 750 thousand acres of farmland. This structure features nine radial gates, each with a width of 5 m and separated by eight piers, each measuring 2 m wide. Lastly, the Abasi regulator, positioned 1054.7 km downstream of AOD and upstream of Zefta Barrage, boasts a remarkable capacity of up to 30 MCM/d, serving 800,000 farm acres. Comprising eight 5-m wide radial gates separated by seven 2-m-wide piers, the Abasi regulator plays a vital role in water management within the region. Table 1 presents.

**Table 1:** Hydraulic characteristics of the employed three radial gates under submerged flow conditions^46^.

Variables/structure	Al-Tawfiki	Al-Menoufi	Abasi
Maximum upstream depth, y_1_ (m)	5.83	5.67	4.74
Maximum downstream depth, y_3_ (m)	4.82	4.79	3.95
Maximum discharge, Q (m^3^/s)	182.29	208.33	189.55
Maximum gate opening, w (m)	1.78	1.64	1.53
Gate radius, r (m)	7.5	8.7	6.03
Pinion height, a (m)	6.95	7	5
Gate width, b* (m)	5	5	5
Number of gates, N	6	9	8
Canal width, B (m)	41	62	54
Number of data	358	361	63

#### Input parameters

**Fig. 1:**
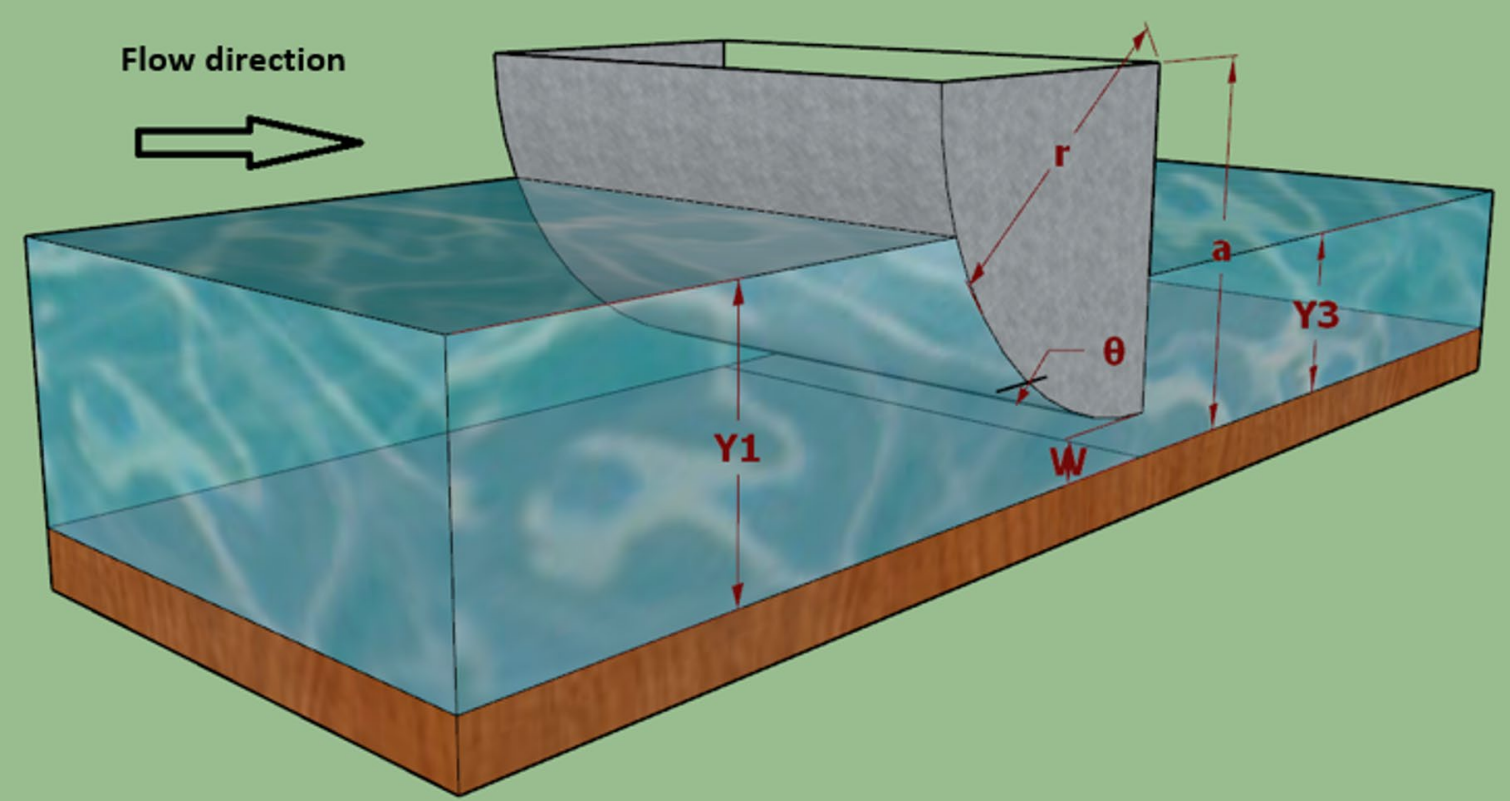
Sketch of radial gates under submerged flow conditions.

Figure 1 shows a sketch of radial gate under submerged flow conditions and the variables that affect the bed configurations downstream of radial gate, which can be expressed in the following functional form:

1 $$f{\text{ }}(Q,{\text{ }}{y_1},{\text{ }}{y_3},{\text{ }}w,{\text{ }}r,{\text{ }}a,q,{\text{ }}b,{\text{ }}B,{\text{ }}{Q_{th}},{\text{ }}g,r,m,d)$$

where the variables are the magnitude of the discharge following under the gate (Q), upstream depth (y_1_), downstream depth (y_3_), canal width (B), total width of open gates (b); gate opening (w), theoretical discharge (Q_th_), gate leaf angle (θ), gate radius (r), pinion height (a), water density (ρ), dynamic viscosity (µ), gravitational acceleration (g) and contraction coefficient (δ). The following dimensionless groups are derived to represent the phenomenon based on dimensional analysis using Buckingham’s theorem:

2 $$\:\mathrm{f}\:(\frac{{y}_{1}}{w},\:\frac{{y}_{3}}{w},\:\frac{r}{w},\frac{Q}{{Q}_{th}}={C}_{d},\frac{a}{w},\frac{b}{w},\frac{\boldsymbol{B}}{w},\frac{Q*{\uprho\:}}{B*{\upmu\:}}={R}_{e},{\uptheta\:},\frac{{y}_{3}}{{y}_{1}},{\updelta\:})=0$$

where Cd is discharge coefficient; Re is Reynolds number. In this study, b, B, g, ρ, and µ, are maintained constant so exclusion is justified due to site uniformity and data availability, while acknowledging possible bias as a limitation, also in open channel flow, the effect of viscus force is negligible with respect to inertia force. Thus the equation does not include them, instead including the coefficients affecting the discharge coefficient, according to^27^, and Cd is defined as:

3 $$\:{C}_{d}=f\left(\right(\frac{{y}_{1}}{w},\:\frac{{y}_{3}}{w},\:\frac{r}{w},\:\frac{a}{w},\:{\uptheta\:},\frac{{y}_{3}}{{y}_{1}},{\updelta\:})$$

**Fig. 2:**
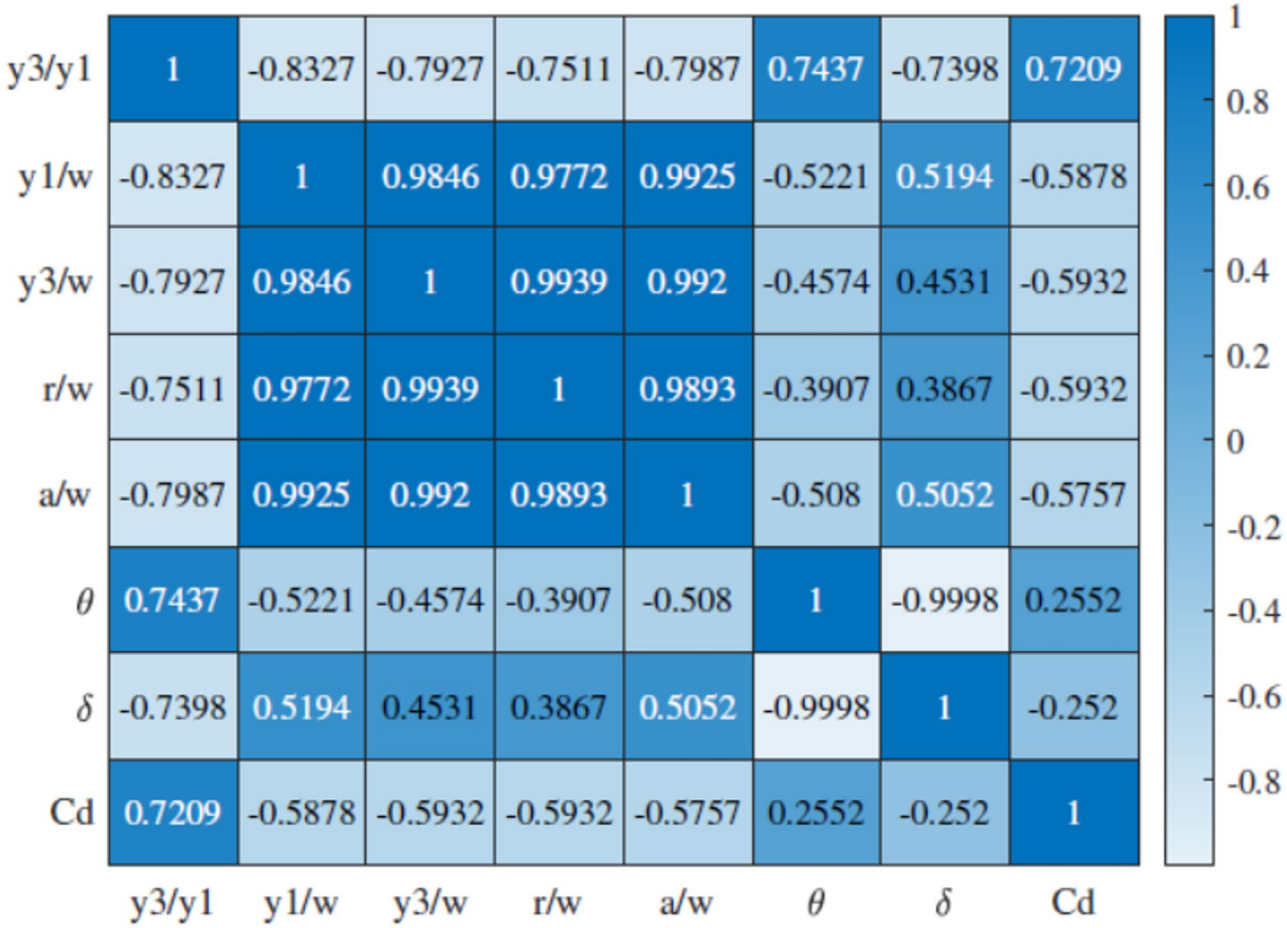
The correlation matrix between all dimensionless input parameters^46^.

Table 2 presents the statistical descriptive analysis of input parameters and C_d_. Besides, Fig. 2 illustrates the correlogram depicting the linear relationship between input and output using the Pearson correlation coefficient. This analysis aids in pinpointing the key input parameters crucial for estimating the C_d_.

**Table 2:** Descriptive statistics for input parameters^46^.

Parameters	y_3_/y_1_	y_1_/w	y_3_/w	*r*/w	a/w	θ	δ	C_d_
Minimum	0.48	2.89	2.56	3.95	3.279	0.476	0.707	0.48
Maximum	0.896	24.57	12.83	39.15	31.5	0.96	0.86	0.98
Mean	0.71	6.93	4.639	10.27	8.775	0.727	0.778	0.695
Skewness	0.25	1.97	1.69	2.018	1.929	− 0.22	0.294	0.66
Kurtosis	2.335	9.05	7.867	9.49	8.885	2.299	2.295	3.07
Coef. of variation	13.86	47.55	33.11	50.54	48.4	14.09	4.24	14.54
Std. deviation	0.098	3.296	1.536	5.19	4.248	0.1	0.03	0.1
Std. error of mean	0.004	0.12	0.06	0.19	0.15	0.004	0.001	0.004
Corr. with C_d_	0.72	− 0.59	− 0.59	− 0.59	− 0.58	0.26	− 0.25	1

Coef., Coefficient; Std., Standard; Corr., Correlation.

**Fig. 3:**
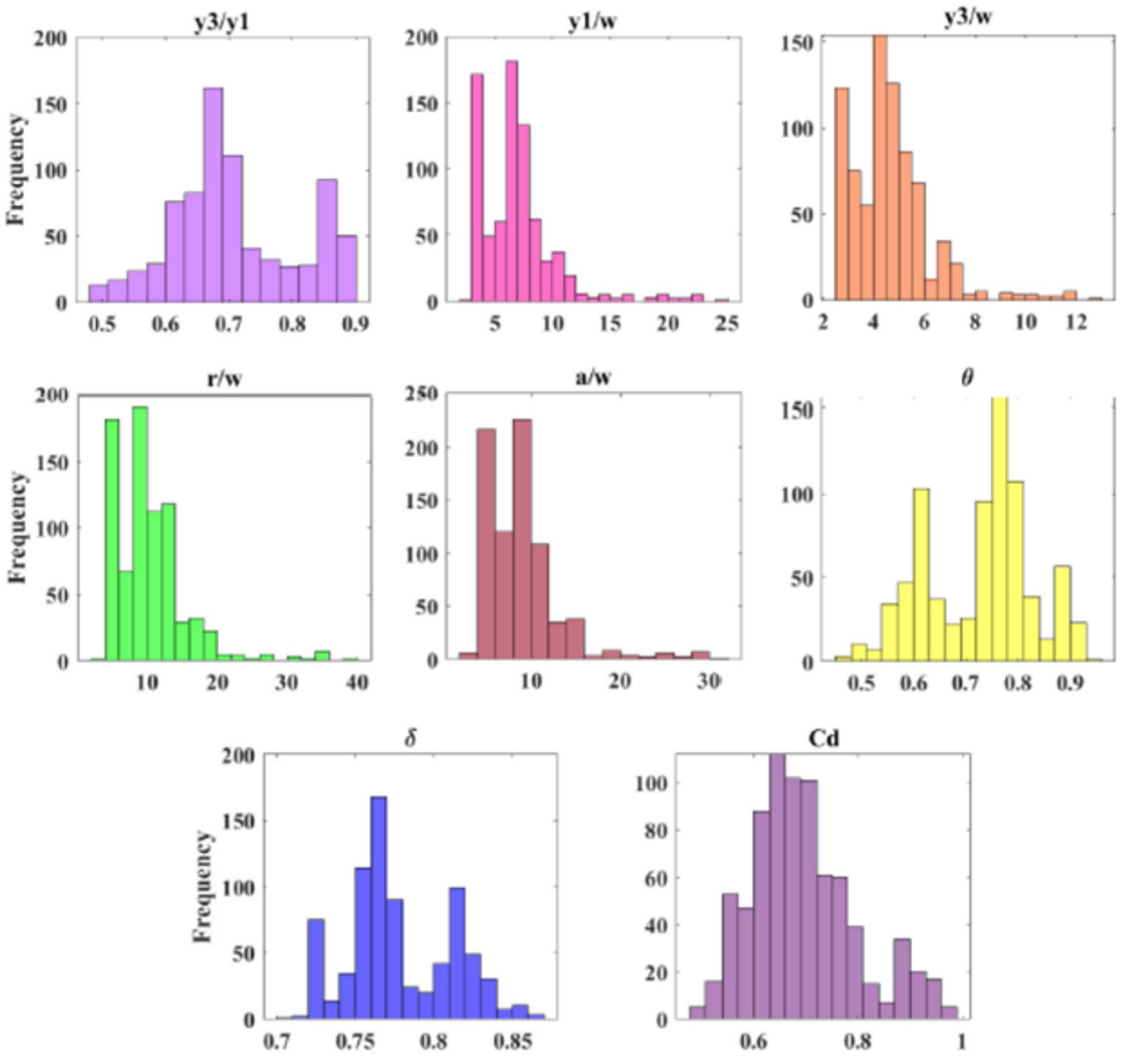
Histogram of dimensionless input variables for submerged flow.

The results revealed that y_3_/y_1_ takes the spotlight and has the highest magnitude of Pearson correlation coefficient (Pc = 0.7209). Therefore, this parameter emerges as the most effective feature for modelling the C_d_ in the submerged radial gates. Moreover, Fig. 3 display histograms of the dimensionless input parameters. These histograms offer concise summaries of the frequency distributions, providing insights into the factors controlling flow discharge in radial gates. Examining these histograms, we observe that y_3_/y_1_, θ and δ have a relatively even frequency distribution (skewness = 0.2465, − 0.2228 and 0.2941). Conversely, the frequency distribution for another dimensionless parameter, r/w displays positive skewness (skewness = 2.0183), with the majority of samples falling within the range of 5 to 15. Similarly, y_1_/w and a/w exhibit an asymmetric frequency distribution (skewness = 1.9685 and 1.9291).

### Ensemble learning

**Fig. 4:**
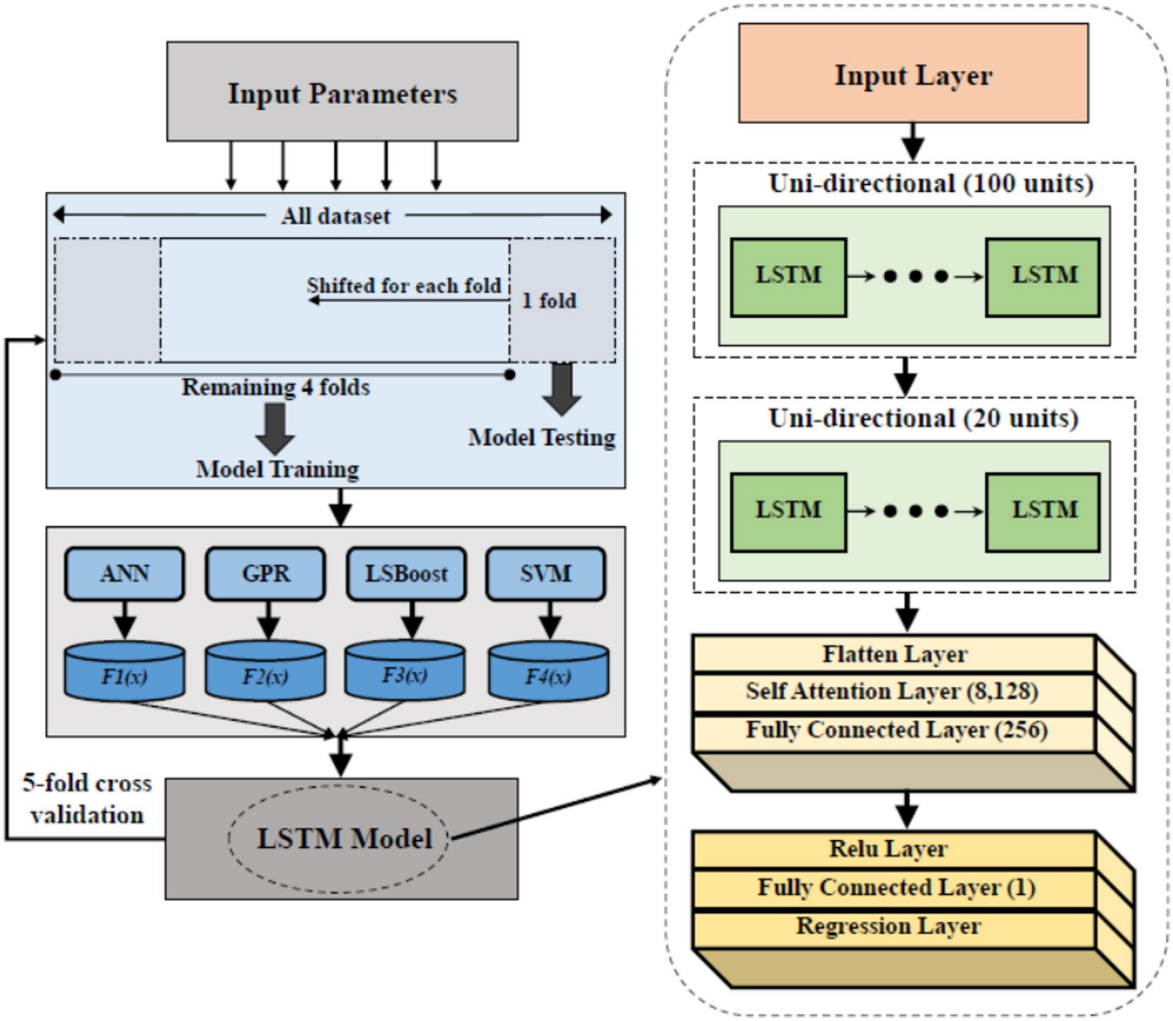
The developed ensemble model architecture.

Ensemble learning stands out as one of the most effective approaches for enhancing system performance. It involves the integration of diverse separate models, working in concert to enhance the stability and predictive prowess of the model^47^. We implemented a stacking technique within our system, called Ensemble. This study adopted a strategy of employing multiple algorithms to train on the same dataset, yielding noteworthy system performance when coupled with stacking. The proposed approach elevates prediction accuracy beyond the capabilities of individual algorithms, exploiting the diverse problem-solving abilities inherent in multiple regression models. Specifically, our ensemble approach incorporated four base models, including GPR, SVM, LSBoost and ANN. The aim was to extract meta-features from each model. Subsequently, these meta-features were inputted into a meta-model, culminating in the final prediction step. Figure 4 displays the proposed ensemble model in this study. The following sections deliver a brief illustration of the proposed approach components.

#### Gaussian process regression

GPR is a potent statistical tool in the field of data-driven modelling. GPR methods offer a non-parametric approach that proves invaluable for tackling a wide range of regression and modelling challenges. Rooted in the Bayesian framework, GPR can be conceptualized as a random process, enabling regression through Gaussian processes^32^. Regarding predictive tasks, GPR stands out as the preferred choice due to its inherent flexibility in providing representations of uncertainty. When considering the function spacef(x) = ϕ$\:{\left(x\right)}^{T}$w and constructing a variable set that conforms to the Gaussian distribution as [f ($\:{x}^{1}$), f ($\:{x}^{2}$), ., f($\:{x}^{n}$)], the GPR model takes the following formulation: f(x) ≈ GP(m(x), k (x, x′)). Where k(x, x′) represents covariance function for input from the training set (x) and testing set (x′) and can be calculated as follows: k(x, x′) = E[(f(x) − m(x))(f(x′) − m(x′))], wherem(x) is the mean function and can be calculated using: m(x) = E[f(x)]. The GPR model output is yielded using: y = f(x)+ϵ, ϵ ≈ N (0, $\:{\sigma\:}_{n}^{2}$). In this context, x represents the input vector, while y corresponds to the output vector. The symbol f denotes the function values within GPR, and ϵ signifies the noise component. GPR models seek to capture patterns within the data relying on a pivotal component, covariance function^41^. Various types of covariance functions can be harnessed as fundamental elements Within the GPR framework including matern32, exponential, ardmatern32, rational quadratic and ardexponential. To effectively train a GPR model, the selection of the appropriate covariance function (k) becomes a critical step as it plays a pivotal role in shaping the actual behaviour of the GPR model. Notably, the geometric structure of the training samples becomes embedded within this function^12^. Ardexponential is utilized in this work.

#### Support vector machines

SVM is renowned as one of the most formidable kernel-dependent models in the prediction domain. This attribute endows SVM with superior generalization and insensitive to over-training. Through the ingenious use of kernel functions, SVM accomplishes the transformation of intricate nonlinear input factors into high-dimensional space, effectively converting complex nonlinear problems into tractable linear ones. Thus, the selection of a kernel function significantly influences the performance of SVM model. The caveat in SVM is based on the quest for an optimal linear or hyper plane, serving as the decision boundary that maximizes the separation margin between the cases. To accentuate, kernel function elegantly addresses the non-linear predictive difficulties within high-dimensional space, standing as one of SVM principal strengths. Within the scope of the present study, delved into the predictive performance of SVM methods employing Radial Basis Function (RBF) kernel. Furthermore, the research optimized of SVM hyper-parameters by methodically conducting a grid search of parameters^28^.

#### Least-squares boosting

LSBoost is another ensemble method involves a large set of weak learners in addition to a meta-learner that sets weights to each learner. The algorithm follows a sequential process through training individual weak learners. Predictions form these learners are combined using voting techniques to ameliorate predictive performance. In particular, the loss criteria in LSBoost is least squares. At each iteration of the process, a new learner is fabricated based on training data to predict the difference between the observed target value and the cumulative predictions from all previously grown learners attempting to correct errors induced by them. Learners are added until the assigned maximum number or the optimal training data error is acquired. This gradient boosting methodology helps to build a durable regression model^45^. In this study, a grid search strategy was applied to the LSBoost method to optimize several parameters, such as learning rate, the number of learning cycles, min parent size, max number of splits and min leaf size, to achieve optimal results. Decision tree was exploited as the weak learner in LSBoost.

#### Artificial neural network

The foundation of ANN model is rooted in the emulation of the human brain function. Much like biological networks, it possesses the capacity to learn and extrapolate from that learning. A pivotal element of biological networks is the neuron, the fundamental building block of the human nervous system. Regarding neural networks, each entity comprises a multitude of nodes connected by directional links, forming a structured arrangement of layers namely, input, hidden, and output. Among the various manifestations of neural networks, back-propagation network is the most prevalent. Multi-layer perceptron (MLP) represents a widely adopted variety within ANN research. The interplay between error signals and input signals fine-tunes the parameters of the MLP. Choosing the number of hidden layers and neurons they contain stands as the most crucial considerations in ANN modeling. For optimal model performance, the sigmoid tangent function was employed for the input layer and the linear function was adopted for the output layer, facilitated by the Levenberg-Marquardt algorithm^43^.

#### Long short-term memory

LSTM architecture comprises input, hidden and output layers. The distinctive feature of LSTM hidden layers is the inclusion of memory cells, each equipped with three gates: input, forget, and output^48^. These gates possess access to both the current input and the previous output. Forget gate assumes the paramount role of determining how much information should be retained or discarded within the memory cell contents. Controlling the forget gate operation involves a neural network with a designated activation function, $\:{f}_{{\uptau\:}}$ = σ (W [$\:{x}_{{\uptau\:}}$, $\:{h}_{{\uptau\:}-1}$,$\:\:{C}_{{\uptau\:}-1}$] + $\:{b}_{\mathrm{f}}$ ). The input gate denoted as $\:{i}_{{\uptau\:}}$ plays a pivotal role in determining what information gets stored in the memory cell. Conversely, the output gate denoted as $\:{o}_{{\uptau\:}}$ decides the timing for transmitting the stored information to the output. The following equations are employed to compute the operations associated with the input and output gates.

4 $$\:{i}_{{\uptau\:}}=\:\left(\mathrm{W}\right[{x}_{{\uptau\:}},\:{h}_{{\uptau\:}-1},\:\:{C}_{{\uptau\:}-1}]\:+\:\:{b}_{\mathrm{i}}\:)$$

5 $$\:{C}_{{\uptau\:}}={f}_{{\uptau\:}}*{C}_{{\uptau\:}-1}+{i}_{{\uptau\:}}*tanh\:\mathrm{W}[{x}_{{\uptau\:}},\:{h}_{{\uptau\:}-1},\:{C}_{{\uptau\:}-1}]\:+\:{b}_{\mathrm{c}})$$

6 $$\:{o}_{{\uptau\:}}=\left(\mathrm{W}\right[{x}_{{\uptau\:}},\:{h}_{{\uptau\:}-1},\:{C}_{{\uptau\:}-1}]\:+\:{b}_{0})$$

7 $$\:{h}_{{\uptau\:}}=\mathrm{tanh}\left({C}_{{\uptau\:}}\right)*{o}_{{\uptau\:}}$$

where b, $\:{C}_{{\uptau\:}-1}$, σ and W denote bias vector, previous LSTM memory, sigmoid function and weights for each input, respectively. As a result, memory cells operate by modulating information through the three controllers: input, forget, and output gates. The forget gate activation function is employed on the previous memory to gauge its relevance in the current memory cell. If the activation output registers as zero, the memory cell will discard the prior information. Consequently, LSTM cells exhibit the capability to retain information over arbitrary durations, selectively controlling the influence of previous time steps^49^.

The proposed LSTM architecture consists of several key components: a sequence input layer for inputting sequences of a specific size, two LSTM layers with 100 and 20 hidden units, flatten layer, attention mechanism and two dense layers with 256 and 1 units using ReLU activation function. The architecture includes a regression layer responsible for producing the predictions.

Table 3 offers detailed properties for each layer. Following the final LSTM layer, the model employs a spatial attention mechanism to enhance its spatial awareness. This is accomplished through the utilization of a multi-head self-attention mechanism, which aids the model in directing its focus towards pertinent spatial regions. The multi-head self-attention process involves partitioning the input data into numerous heads, each of which signifies a unique subspace within the feature space. Within each head, the input is transformed into three distinct spaces: Key (k), Query (Q), and Value (V). Subsequently, attention scores (A) are computed using a scaled dot-product attention mechanism, A= SoftMax( $\:\frac{Q*\:{K}^{T}}{\sqrt{{d}_{k}}}$ ). Here, $\:{d}_{k}$ represents the dimensionality of the key vectors. Next, the scaled attention scores play a crucial role in deriving the ultimate attention output, O = A × V. These outputs from all attention heads are amalgamated and subjected to linear transformation to generate the final attention result. Subsequently, the resulting feature maps are flattened and passed through two fully connected layers, featuring ReLU activation.

**Table 3:** Details of the proposed meta model.

Layers	Type	Properties
Layer 1	Sequence input	1 dimension
Layer 2	LSTM	100 units/sequence-to-sequence
Layer 3	LSTM	20 units/sequence-to-last
Layer 4	flatten	–
Layer 5	Attention mechanism	8 heads, 128 channel
Layer 6	Fully connected	256 units, Relu
Layer 7	Fully connected	1 unit
Layer 8	Regression	Predictions

#### Stacking ensemble model for discharge coefficient prediction

A two-stage stacking ensemble framework was developed to accurately predict the discharge coefficient $\:\left({C}_{d}\right)\:$based on a set of dimensionless hydraulic and geometric input features, including $\:{y}_{3}/{y}_{1}$, $\:{y}_{1}/w$, $\:{y}_{3}/w$, $\:r/w$, $\:a/w$, $\:\theta\:$, and $\:\delta\:$.

In the first stage, multiple heterogeneous base learners—namely Gaussian Process Regression (GPR), Support Vector Machine (SVM), Least Squares Boosting (LSBoost), and Artificial Neural Network (ANN)—were independently trained using the same input feature set. Each base model generated an individual prediction of the discharge coefficient, which collectively formed a meta-feature vector. This vector encapsulates the diverse learning behaviors and predictive strengths of the underlying models.

In the second stage, the meta-feature vector was treated as a short sequential input and passed to a meta-learner designed to perform adaptive model fusion. The meta-learner integrates a multi-head attention mechanism with Long Short-Term Memory (LSTM) layers, followed by fully connected layers. The attention mechanism dynamically assigns weights to the base model predictions using scaled dot-product attention, thereby emphasizing more informative predictions under varying hydraulic conditions. The resulting attention-weighted representation was then processed by the LSTM to capture inter-model dependencies before being mapped to the final discharge coefficient estimate through fully connected layers.

This hierarchical learning strategy enables both robust feature extraction and dynamic weighting of base learners, leading to improved predictive accuracy and generalization capability. The final output of the framework is the predicted discharge coefficient $\:{\widehat{C}}_{d}$.

### Model development and evaluation

In this investigation, five-fold cross validation was used to gauge the performance of the implemented methods. Input data was partitioned into 80/20 subsets for the purposes of training and testing. Precisely, 80% of the dataset was designated for training, leaving the remaining 20% for assessing the model performance. To guarantee an impartial analysis and dependable outcomes, we conducted each experiment five times, deriving the prediction average from these iterations. The development of the proposed models was executed within MATLAB (2022b), and all experimental runs were carried out on a computing device equipped with an Intel Core i7 CPU (3.20 GHz), 16 GB of RAM, and a GTX 1660 Ti GPU. The evaluation metrics employed for model performance assessment encompass root mean square error (RMSE), correlation coefficient (R), Nash Sutcliffe efficiency (NS), Willmott’s agreement index (WI) and mean absolute percentage error (MAPE). These indicators facilitate a comprehensive comparison and analysis of simulation results across different models. These measures are calculated using the following mathematical equations:

8 $$\mathrm{RMSE}=\:\sqrt{\frac{1}{N}\sum\:_{i=1}^{N}{({p}_{i}-{o}_{i})}^{2}}$$

9 $$\:\mathrm{R}=\frac{\sum\:_{i=1}^{N}({p}_{i}-\stackrel{-}{p}\:\left)\right({o}_{i}-\underset{\_}{o}\:)}{\sqrt{\sum\:_{i=1}^{N}{({p}_{i}-\overline{p})}^{2}\:\sum\:_{i=1}^{N}{({o}_{i}-\overline{o}\:)}^{2}}}\:-1\:\le\:R\le\:1$$

10 $$\:\mathrm{N}\mathrm{S}\hspace{0.17em}=\hspace{0.17em}1-\left(\frac{\sum\:_{i=1}^{N}{({p}_{i}-{o}_{i})}^{2}}{\sum\:_{i=1}^{N}{({o}_{i}-\overline{o}\:)}^{2}}\right)\:-\:{\infty\:}\:\le\:\:\mathrm{N}\mathrm{S}\hspace{0.17em}\le\:\hspace{0.17em}1$$

11 $$\:\mathrm{M}\mathrm{A}\mathrm{P}\mathrm{E}\:=\:\frac{100}{N}\sum\:_{i=1}^{N}\frac{\left|{o}_{i}-{p}_{i}\right|}{{o}_{i}}$$

where$\:{\:o}_{i}$ represents the observed values, $\:{p}_{i}$ signifies the predicted values, $\:\overline{o}$ stands for the average of the observed values, and N denotes the total number of observations.

This study, introduced several robust approaches, including four base machine learning models and a meta deep learning model, which together form the ensemble model. Moreover, four longstanding regression models were exploited to validate the performance of the proposed model. Table 4 provides a comprehensive list of all hyperparameters for these models. Regarding the LSTM model, Table 3 highlights the details of each layer. To enhance reproducibility, all predictive models were tuned using a deterministic grid-search procedure. For each repetition, the dataset was evaluated using 5-fold cross-validation. Inside each fold, the training subset was further split into 90% for training and 10% for validation to monitor overfitting and select hyperparameters. Hyperparameter selection was based on the lowest mean validation RMSE, and when multiple configurations achieved statistically similar RMSE (difference ≤ 1% of the best RMSE), the simpler configuration was selected to improve generalization and reduce computational cost. For the SVM model, the grid search explored BoxConstraint (1, 10, 50, 100, 200, 500) and KernelScale (0.1, 0.5, 1, 2.2, 5, 10), with standardization enabled. The final selected values were Box Constraint = 100 and Kernel Scale = 2.2. For LSBoost, the tuned parameters included the number of learning cycles (100, 200, 300, 500, 800), learning rate (0.05, 0.10, 0.20, 0.30, 0.40), maximum number of splits (50, 100, 200, 300), and minimum leaf size (1, 5, 10, 20). The final configuration was Learning Cycles = 500, Learning Rate = 0.3, MaxNumSplits = 300, and Leaf Size = 10.

For the ANN model, we tested one hidden layer with neurons (20, 40, 60, 80, 100, 120), learning rate (10^−4^, 5 × 10^−4^, 10^−3^, 5 × 10^−3^), and epochs (50, 100, 150, 200). The final configuration used 80 neurons, LR = 0.001, and 100 epochs.

For GPR, several kernel functions were evaluated, including (exponential, squared exponential, matern32, matern52, rationalquadratic, ardexponential), and the kernel yielding the best validation RMSE (ardexponential) was retained as the final kernel.

For the LSTM model, we performed a two-stage grid search (coarse then refined around the best region) to avoid an excessively large search space. The explored parameters were: hidden units in the first LSTM layer (50, 100, 150), hidden units in the second LSTM layer (10, 20, 30), mini-batch size (64, 128, 256), learning rate ({10^−4^, 5 × 10^−4^, 10^−3^, 2 × 10^−3^), attention heads (4, 8), and attention channel size (32, 64, 128). Training employed Adam optimization with a maximum of 1000 epochs. The final architecture used 100 and 20 hidden units, mini-batch size = 256, learning rate = 0.001, and multi-head attention with 8 heads and 128 channels.

On the stated workstation, the average training time per fold was approximately: SVM 0.3–0.7 s, GPR 0.8–1.6 s, LSBoost 2–5 s, ANN 3–8 s, and the LSTM-attention meta-model 8–20 s. Overall, the complete training and evaluation of the stacked ensemble across 5 folds required approximately 6–12 min, which was considered acceptable given the improvement in predictive accuracy and robustness. Importantly, configurations with higher complexity (e.g., > 150 LSTM units or > 8 attention heads) yielded negligible RMSE gains while increasing training time by ~ 25–60%, and therefore were not adopted.

**Table 4:** Optimal configurations for all the models.

Model	Parameter settings
ANN	1 hidden layer, No. of neurons = 80, LR = 0.001, Epochs = 100
LSBoost	Splits = 300, Split Criterion = mse, Leaf Size = 10, LR = 0.3, Learning Cycles = 500
GPR	Covariance function: Ardexponential
SVM	Kernel Function = RBF, Kernel Scale = 2.2, Box Constraint = 100
Bagging	Splits = 100, Split Criterion = mse, Leaf Size = 10, Learning Cycles = 200
GLM	Link function = Inverse gaussian
GRNN	Spread value = 0.01
RNN	2 hidden layers, No. of neurons = [20 20], Activation function = Relu

## Results

The proposed ensemble model achieved the best overall predictive accuracy among all developed models, yielding RMSE = 0.0175, *R* = 0.9843, NS = 0.9684, and WI = 0.9918, which confirms its strong agreement with the observed (Cd) values. Among the standalone/base learners, GPR delivered the most competitive performance (RMSE = 0.0183, *R* = 0.9829, NS = 0.9657, WI = 0.9912) and also produced the lowest MAPE (1.3112%), indicating superior relative-error behavior. The remaining base models—RNN, SVM, and ANN—also provided accurate estimates, with RMSE values of 0.0194, 0.0195, and 0.0199, respectively. In contrast, the traditional regression benchmarks exhibited weaker performance, where GLM produced the largest error (RMSE = 0.0417, MAPE = 4.9187%), followed by Bagging and GRNN with RMSE values of 0.0334 and 0.0355, respectively.

**Fig. 5:**
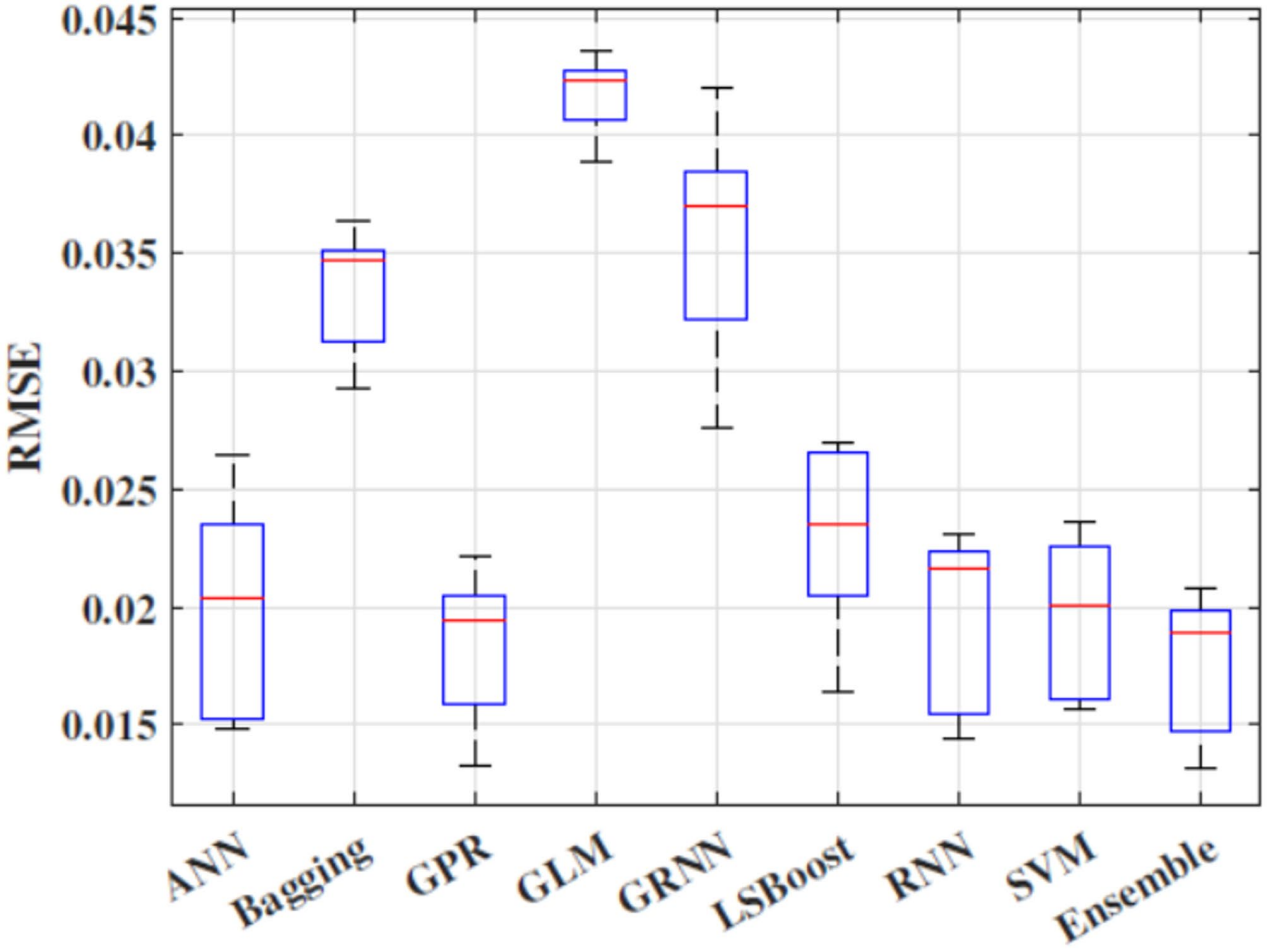
RMSE performance for all the implemented models across 5-fold cross validation set.

**Fig. 6:**
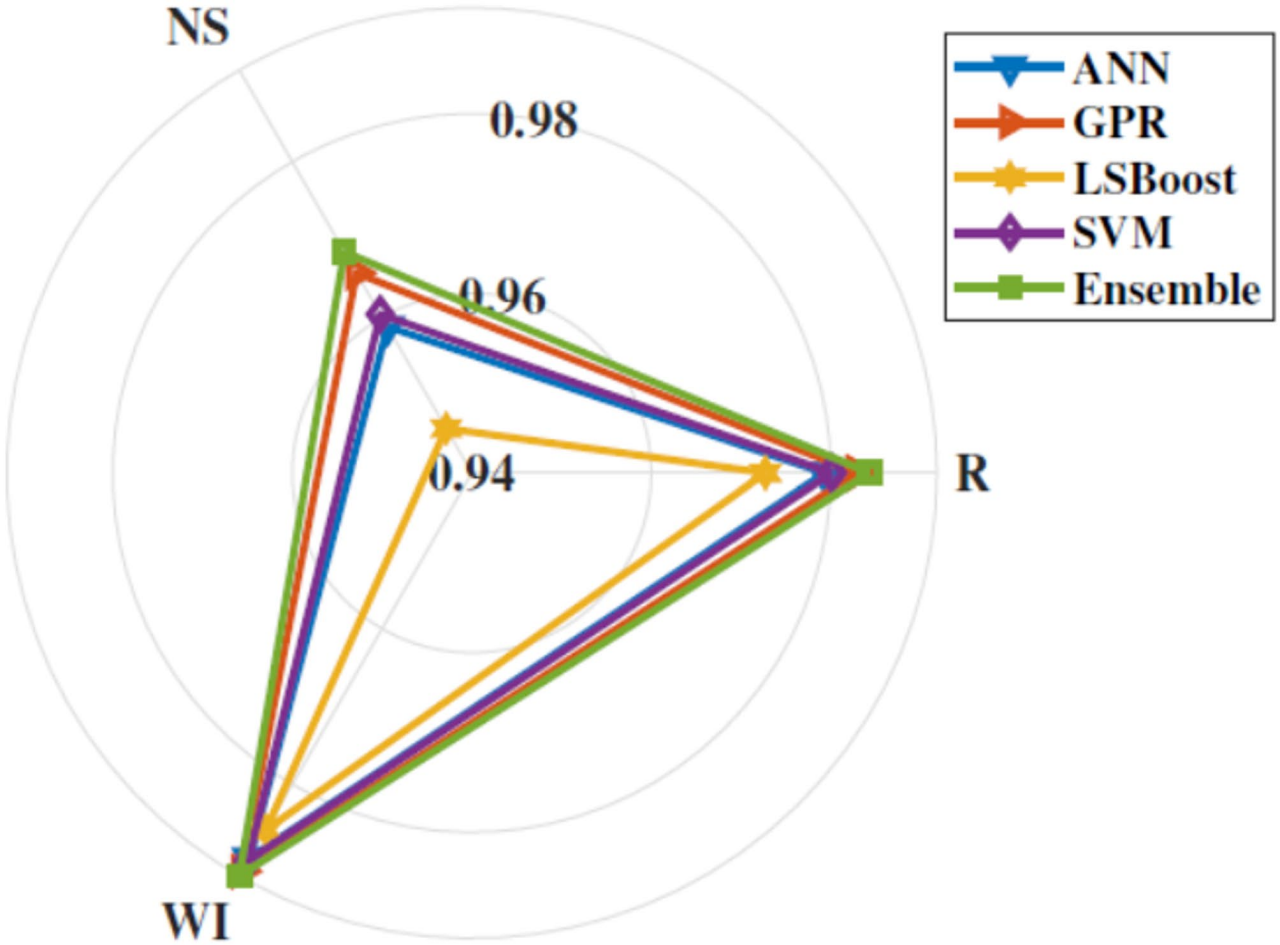
Comparison results between the proposed ensemble and base models.

**Fig. 7:**
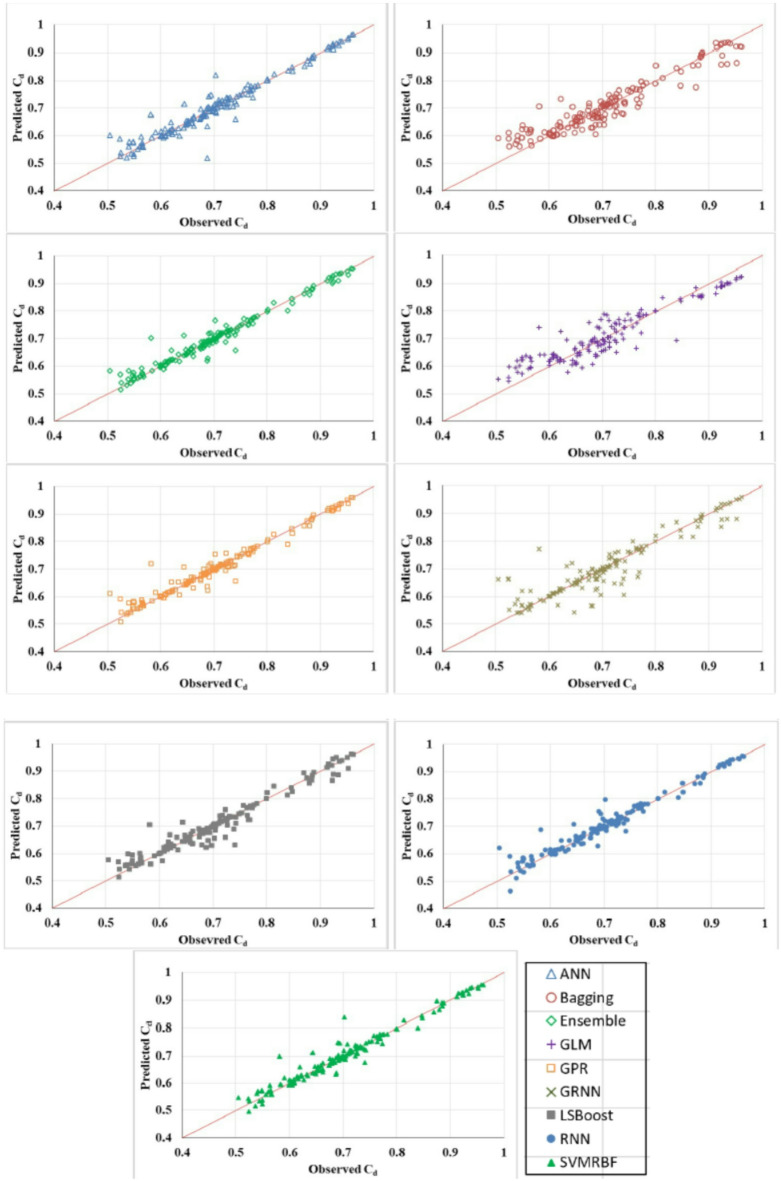
Plot of observed and predicted C_d_ using all the implemented models.

**Fig. 8:**
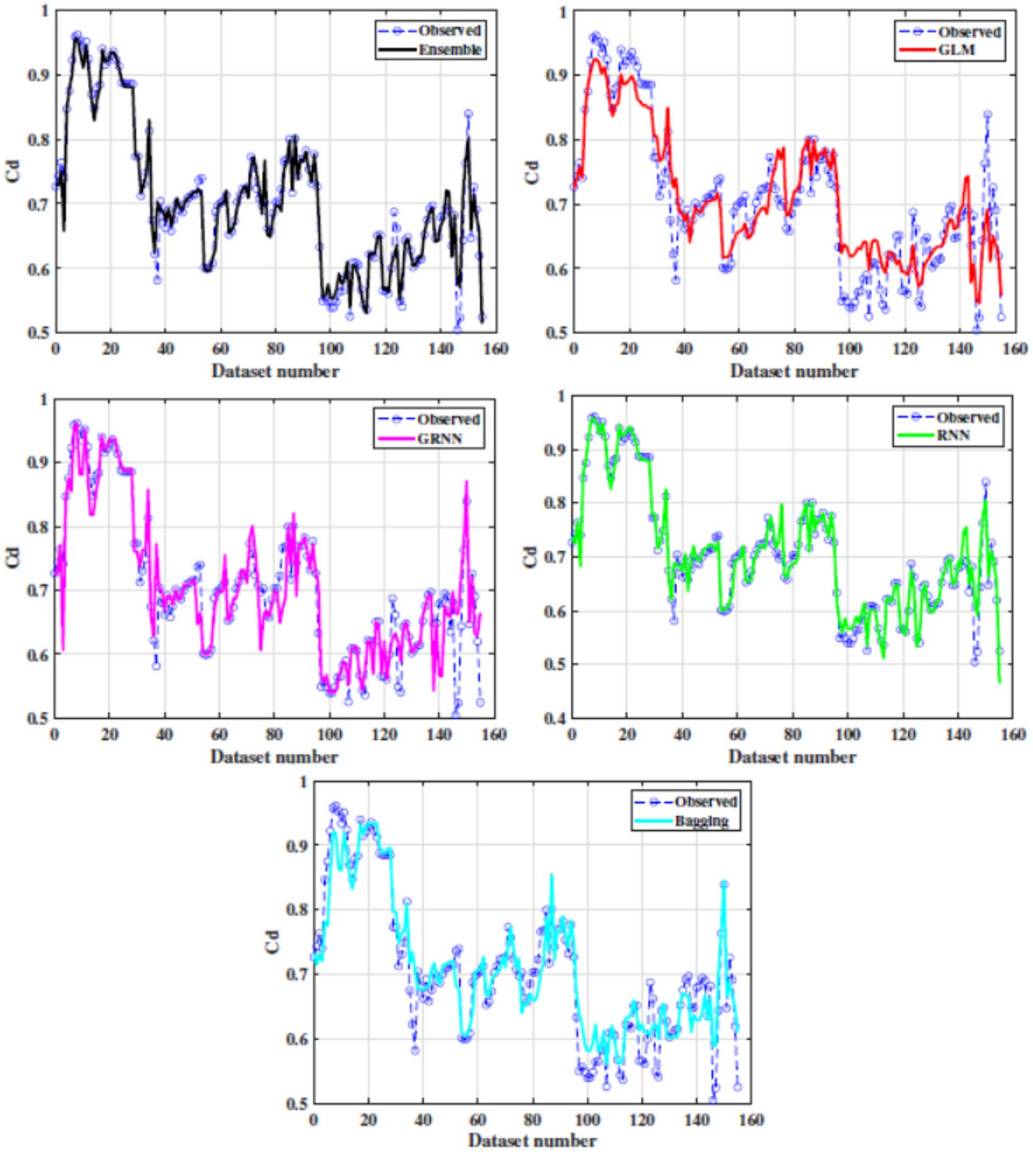
Performance plot between observed and predicted C_d_ by ensemble and existing models.

Figure 5 shows box plot of RMSE of all the implemented models. These results suggest that the Ensemble model demonstrated superior efficiency and accuracy across the five folds compared to other models. This is evident from its favourable performance in terms of RMSE, surpassing the base and traditional models. Additionally, the statistical results in Fig. 6 indicate that the proposed Ensemble model outperforms the base models in terms of R, NS, and WI. Exceptionally, the ensemble model estimated C_d_ values closely aligned with the observed range, outperforming other models. Figure 7 presents agreement plots for all the proposed methods. Using an ensemble, the observed data points closely align with the y = x line, signifying a strong correspondence (i.e. the predicted C_d_ values generated by the ensemble model closely align with the ideal 45-degree line). However, in the case of the GLM method, the data points exhibit notable deviation from the y = x line, characterized by considerable fluctuations in the datasets. It demonstrates that the stacked ensemble model outperforms the other implemented models. In Fig. 8, the performance plots compare predictions from the proposed model to those from other longstanding regression models. A consistent alignment between Ensemble predictions and the actual measurements is clear. These visualizations provide strong evidence that the ensemble model stands out as the optimal choice for C_d_ values prediction.

**Fig. 9:**
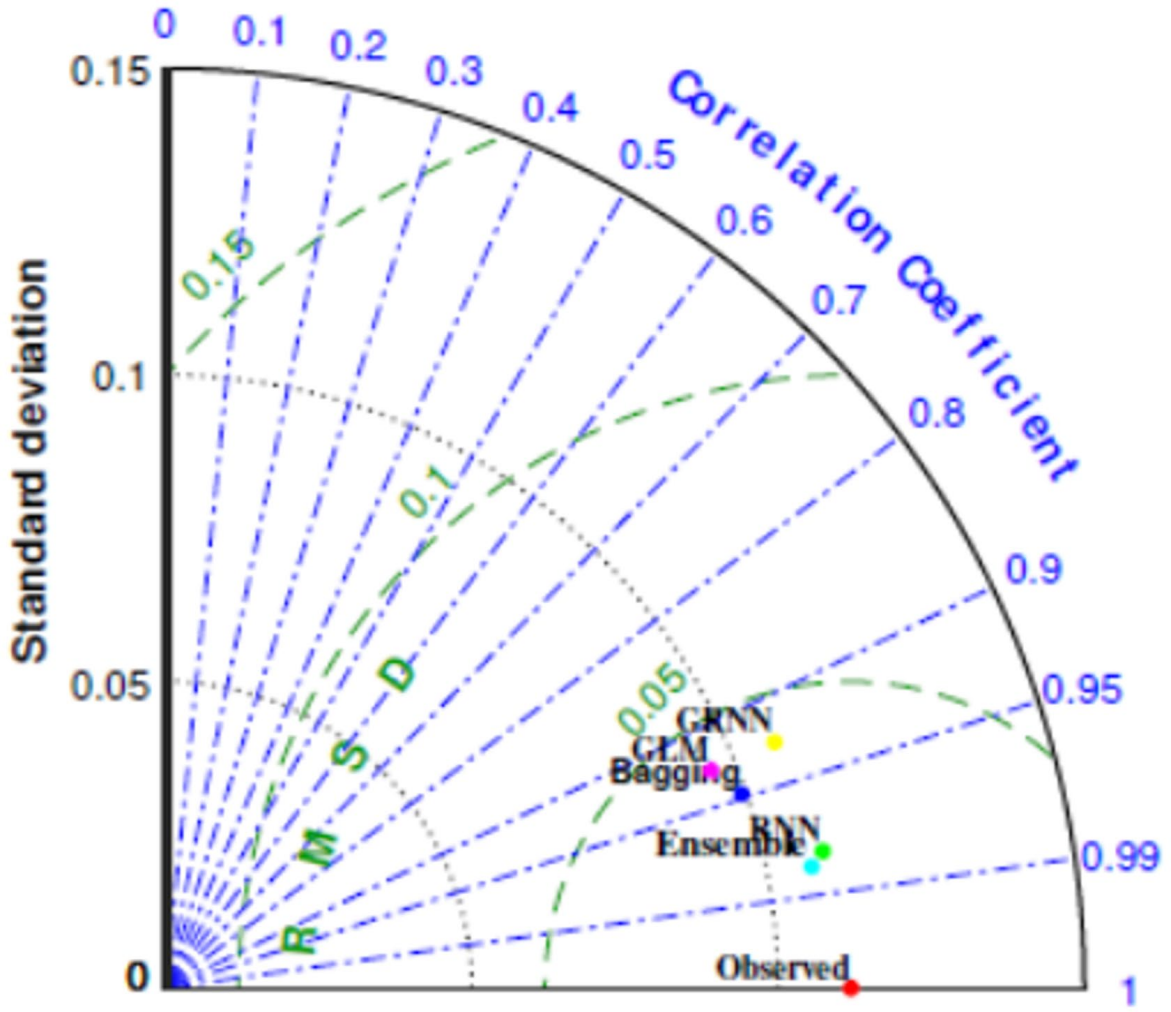
Taylor’s diagram for comparing the performance of the proposed ensemble and traditional methods.

**Fig. 10:**
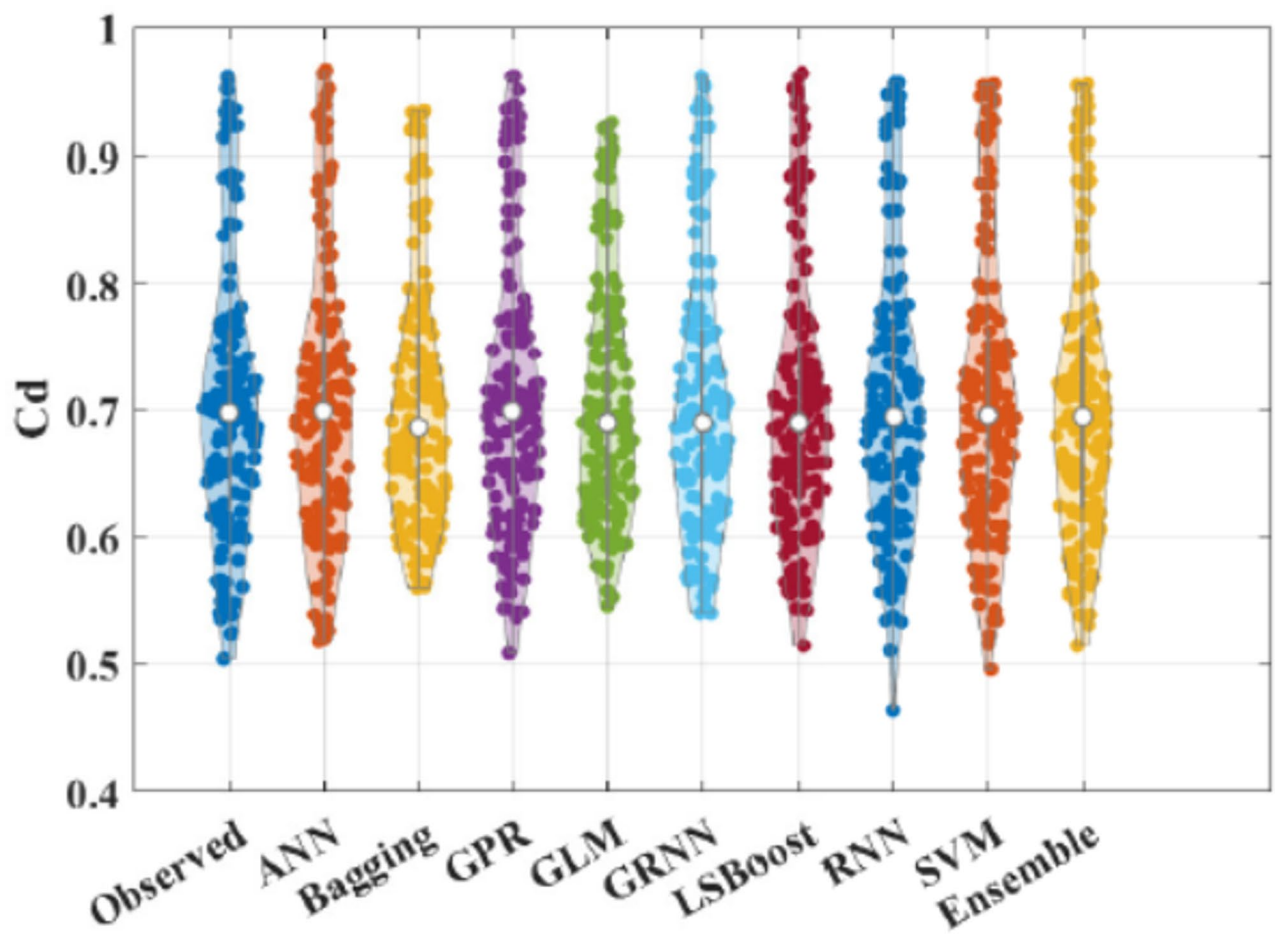
Violin plots of observed and estimated C_d_ using all the models.

Figure 9 offers another comparative evaluation of the performance of the ensemble model along with four existing regression methods through Taylor’s diagram (TD), which encompasses three key statistical parameters, standard deviation, R and RMSE. This analysis employs TD to schematically assess the accuracy of the models. The optimal model is the one demonstrating the highest correlation (*R* = 1), positioned near the red circular symbol along the X-axis. This figure reveals that ensemble is in closer proximity to the red reference point of observed data compared to Bagging, GLM and GRNN, obtaining higher correlation with diminishing RMSE value. RNN exhibits good performance, as evidenced by its comparable distance from the red reference point of observed data. Consequently, Ensemble and RNN demonstrate success in C_d_ prediction during the testing phase. Moreover, to evaluate the performance of all the models in estimating C_d_, a violin plot is utilized. This type of plot combines elements of box plot with kernel density plot. The violin plots represent both observed and estimated values of C_d_, generated by the ensemble and all other models. Figure 10 distinctly illustrates that the C_d_ estimation by the ensemble model and GPR closely mirrors.

### Sensitivity analysis

The prediction accuracy for the submerged flow scenario displayed certain limitations. This can be attributed to the inherent complexity of phenomena in such scenario. Accordingly, the need for input variables to fully grasp their physical significance is crucial. In this work, input parametrs, y_3_/y_1_, y_1_/w, y_3_/w, r/w, a/w, θ and δ are employed. While, C_d_ is greatly affected by various physical parameters, given that flow discharge is linked to numerous hydraulic variables. Therefore, conducting sensitivity analysis to pinpoint the parameters that impact C_d_ magnitude is a pivotal step for such modelling process. In this sensitivity analysis, we systematically excluded each input parameter and assessed the impact on the ensemble model using statistical criteria. Then, the results were compared to the original situation where all variables were present. Table 5 provides statistical measures for the employed parameters using the optimal predictive model. Notably, y_3_/y_1_ emerges as the influential variable in C_d_ calculations. This result is consistent with a previous study^12^. Also, y_3_/w is ranked second and proved to have a significant impact on C_d_ predictions.

**Table 5:** Sensitivity analysis for input parameters using the proposed ensemble model.

Metrics	All—y_3_/y_1_	All—y_1_/w	All—y_3_/w	All—*r*/w	All—a/w	All—θ	All—δ	All
RMSE	0.0196	0.0176	0.0185	0.0181	0.0177	1.5547	0.0177	0.0175
R	0.9805	0.9841	0.9825	0.9832	0.9841	0.9840	0.9841	0.9843
NS	0.9606	0.9680	0.9648	0.9662	0.9678	0.9677	0.9677	0.9684
WI	0.9897	0.9917	0.9908	0.9912	0.9916	0.9916	0.9916	0.9918
MAPE	1.7578	1.5160	1.6595	1.5619	1.5547	1.5493	1.5346	1.5260
Rank	1	7	2	3	4	5	6	–

From a hydraulic perspective, the sensitivity results are physically consistent with submerged radial-gate flow behavior. The ratio y_3_/y_1_ represents the degree of submergence (tailwater influence relative to upstream head) and therefore governs the reduction in effective head across the gate and the extent to which the downstream water level suppresses jet development and modifies contraction beneath the gate. As y_3_/y_1_ increases, the submerged jet becomes increasingly controlled by tailwater, which directly alters the discharge coefficient C_d_. This explains why removing y_3_/y_1_ causes the largest degradation in prediction accuracy. The second most influential term, y_3_/w, reflects the downstream depth relative to the opening and is linked to submerged jet thickness and contraction conditions, which also control energy dissipation and the resulting discharge behavior. In contrast, the geometric terms primarily influence the effective flow passage and streamline curvature, leading to smaller, but still non-negligible, effects on C_d_, while δ captures contraction-related influences associated with local gate conditions. These findings provide practical operational guidance for submerged-gate discharge estimation: (i) accurate and stable measurement of both upstream and downstream water levels is essential because errors in y_3_/y_1_ propagate strongly into C_d_ and discharge estimates; (ii) calibration and real-time discharge computation should explicitly track variations in tailwater submergence, particularly under high-submergence operating conditions where C_d_ ​ is most sensitive; and (iii) periodic inspection/maintenance actions that affect contraction behavior can improve reliability of discharge estimation, even when stage measurements are accurate.

## Discussion

Irrigation projects commonly rely on radial gates for precise discharge measurements, a critical component in effectively distributing water to end-users. These radial gates aid in controlling flow, eliminating the need for separate flow measurement structures^5^. However, achieving accurate flow measurement poses several shortcomings, primarily attributed to structural variations^11^. Furthermore, gates operating under submerged flow conditions often encounter high error rates in calibration^12^. Accordingly, the primary motivation behind this study is to develop a robust model for automatic prediction of C_d_. Radial gates C_d_ estimation has received limited attention in the existing literature, leaving a significant gap that this study aims to address. To bridge this gap, we first evaluated several well-established predictive models known for their strong performance, including ANN^28,43,50^, Bagging, GLM^1,19,43^, GRNN^4^, RNN, LSBoost^45^, GPR^1,12,41^ and SVM^32,42^. These models were tested on a diverse dataset sourced from three different canals. The dataset comprised 782 field data points under submerged flow conditions, providing a solid foundation for constructing accurate predictive models. This aligns with study objective of developing a reliable modeling algorithm. Onward, we developed a stacking ensemble model incorporating the best-performing predictive models as base models, with an LSTM model enhanced by an attention mechanism serving as the meta-model for predicting C_d_. Moreover, we conducted a comparative study between the ensemble model and the base models, as well as various regression models from previous studies, to assess our model predictive performance.

A direct comparison with state-of-the-art models from previous research on C_d_ prediction was conducted to provide a comprehensive assessment of the overall performance of the proposed method. Authors in^12^ exploited GPR with ardmatern32 kernel function to yield R of 0.997. In another study, Roushangar et al.^32^ utilized SVM model for predicting C_d_ in a radial gate under submerged conditions, while securing a maximum R of 0.940 and RMSE of 0.022. Norouzi et al.^43^ reported that multiple models driven by ANN is the most outperformed standalone models for C_d_ estimation with R of 0.784 and RMSE of 0.087. The performance of the proposed model was assessed by different statistical measures using 5-fold cross validation technique. Our ensemble model achieved RMSE, R, NS and WI of 0.0175, 0.9843, 0.9684 and 0.9918. While, the base models achieved RMSE between 0.0230 and 0.0183 and R between 0.9727 and 0.9829.

On the other hand, the existing models yielded RMSE between 0.0417 and 0.0194 and R between 0.9098 and 0.9804. Notably, the comparison between descriptive statistics of all the models revealed that the GPR model outperformed all the employed models in terms of MAPE. It is worth noting that GPR, as previously reported in^1,12^, have been identified as effective model capable of predicting C_d_ accurately for submerged radial gates. In parallel, SVM is known for delivering superior performance compared to other regression models^32^. Both techniques yielded RMSE of 0.0183 and 0.0195, respectively. These results signify that the proposed stacked model exhibited remarkable improvement in the performance when compared to the seasoned standalone models for C_d_ prediction. Moreover, the prior results confirm that the proposed model exhibits improved predictability performance when compared to the aforementioned literature studies. Isolated assessment of individual regression models does not necessarily reveal the most effective combinations of them owing to significant interactions. Also, the addition of more models may not necessarily ameliorate prediction accuracy. As the number of base models increases, optimizing weights may not significantly outperform simple averaging^51^. In fact, using too many base models can potentially reduce the ensemble model performance^47^. Therefore, it was imperative to convey a thorough search to identify the most pivotal combinations of base models, as emphasized in^43^. Also, it is important to recognize that both GPR and SVM can deliver highly accurate predictions, particularly under submerged flow conditions, making them challenging benchmarks to surpass^12,32,41^. GPR outperformed extreme learning machine model with grey wolf optimization kernel as reported in^32^, while SVM betters multigene genetic programming model in^12^. The proposed stacking ensemble model introduced demonstrated a notable improvement compared to these techniques, affirming the validity of the proposed approach. This study underscores the potential for enhancing prediction performance through the fusion of diverse ML models with DL approach. The strength of the proposed model lies in the ability to dynamically learn the weights assigned to each base model. This adaptability enables it to harness the strengths of individual base models more effectively. The developed LSTM model with attention mechanism helped to flexibly adjust the contribution of each base model based on the specific characteristics of different data points. In contrast, traditional approaches may offer only limited improvements in overall performance. For instance, authors in^52^ implemented a stacking model for rainfall prediction, however ANN model outperformed their stacking model. This discrepancy primarily stemmed from most ML models underestimating extreme precipitation points. In particular, the models in^43,52^ did not assign sufficient weight to each base model. Furthermore, in contrast to the ensemble learning approach by^51^, the LSTM model possesses the capability to assign weights to the output of base models, effectively emphasizing crucial features. This strategic approach mitigates the effect of lavish noise and irredundant features on the meta model, ultimately enhancing both model stability and accuracy. These findings suggest that the proposed approach, which incorporates stacking ensemble learning with LSTM and an attention mechanism, significantly enhances the system accuracy and performance.

## Implications

The proposed stacking ensemble model offers practical value for improving discharge estimation and operational decision-making in submerged multi-parallel radial gates. Beyond reporting predictive accuracy, this section discusses how the model can be deployed in real gate-control environments, its expected behavior under extreme regimes, and key limitations that guide safe application and future extensions.

### Integration into gate operation and calibration workflows

In practical irrigation systems, discharge estimation is typically required for (i) real-time gate operation (adjusting openings to meet target deliveries), and (ii) periodic calibration and performance auditing of regulators. Because the proposed system predicts (Cd) directly from dimensionless hydraulic and geometric variables, it can be integrated as a lightweight decision-support layer on top of existing SCADA or telemetry-based monitoring. A feasible deployment workflow is as follows: (1) upstream and downstream water levels are obtained continuously from level sensors, while gate opening and gate angle are obtained from actuator position sensors; (2) the ratios are computed in real time, while geometric terms and use known gate parameters and measured; (3) the ensemble model returns an updated (Cd), which is used to compute discharge through standard submerged-gate discharge relations; and (4) the predicted discharge is compared against target setpoints and operational constraints to adjust gate openings. For calibration tasks, the model can be used to rapidly generate updated (Cd) estimates under different submergence conditions and detect deviations from expected patterns that may indicate structural or maintenance issues. This approach is particularly useful in settings where submerged-flow calibration is known to be uncertain, and where operators require a robust, data-driven alternative to complex curve-based procedures. The ensemble architecture is also beneficial operationally: the meta-learner (attention-based LSTM) adaptively weights the base learners, which helps provide stable predictions across varying hydraulic states.

### Behaviour under extreme or extrapolative regimes

Like most data-driven models, reliability is highest when the operating conditions fall within the range represented in training data. The dataset used in this study represents real field conditions from three regulators under submerged flow, with variable ranges summarized in Table 2. Therefore, the model should be expected to perform best within comparable ranges of submergence ratio and opening-depth ratios. Under extreme or extrapolative regimes (e.g., unusually small gate openings, very high submergence, or operating combinations not observed in the dataset), prediction uncertainty may increase and may lead to biased (Cd) estimates. To support safe deployment, we recommend incorporating simple “validity checks” before using predictions for automated control: (i) monitor whether input ratios fall outside the observed bounds (Table 2) and flag the prediction as extrapolative; (ii) switch to conservative fallback rules (e.g., conventional calibration curves or safety-limited discharge computation) when extrapolation is detected; and (iii) continuously log operational data to enable periodic retraining or updating of the model as new extreme events are observed. These safeguards are straightforward to implement and are common practice when deploying machine-learning models in operational engineering systems.

### Limitations and future directions

Despite its high predictive performance, the proposed system has several important limitations that should be acknowledged for transparent interpretation and responsible use:

1. The model was trained using field measurements from three regulators in the Nile Delta system. While these data enhance practical relevance compared with laboratory-only studies, hydraulic behavior can vary across regulators due to differences in geometry, approach conditions, downstream channel transitions, maintenance state, and local energy losses. Consequently, direct transfer to other regulators may lead to performance degradation unless local data are incorporated.
2. Real-world sensors may be affected by noise, drift, or intermittent failures. Since the most influential variable identified in sensitivity analysis is the submergence ratio, errors in level measurements may propagate into Cd estimates. Similarly, missing measurements of (w) or (θ) can disrupt computation of dimensionless inputs. In practice, robustness can be improved through (i) filtering or smoothing of level measurements, (ii) routine sensor validation and redundancy (e.g., dual level sensors), and (iii) simple imputation strategies for short periods of missing data (e.g., last observation carried forward for stable conditions), while flagging such estimates for caution.
3. Although the proposed stacking strategy is generic, applying it to other hydraulic structures requires reconsideration of the governing dimensionless inputs and structure-specific parameters. A promising direction is transfer learning, where the meta-model is retained and fine-tuned using a limited set of new site-specific data. Another practical pathway is to train a generalized model using multi-site datasets (multiple regulators and channel configurations), which would expand applicability and improve robustness under a wider range of hydraulic conditions.
4. Rare operating regimes (e.g., extreme tailwater conditions) are underrepresented in typical operational datasets. Future work should incorporate broader field measurements across seasons and events, and evaluate the model under stress-testing scenarios. Incorporating uncertainty estimation (e.g., prediction intervals from base learners such as GPR) could provide additional operational confidence when the model is used for real-time decision support.

Overall, the proposed ensemble model is well-suited as a decision-support tool for submerged radial gate discharge estimation, especially within the operating ranges represented by the collected field dataset. Future developments should prioritize multi-site expansion, robustness under noisy/missing data, and adaptation strategies to facilitate transfer to other regulators and hydraulic structures.

## Conclusion

This study presented a novel deep learning-based stacking ensemble framework for predicting the discharge coefficient (Cd) of submerged multi-parallel radial gates using large-scale field data from three operational irrigation regulators in Egypt. Unlike conventional empirical formulations and standalone machine learning approaches, the proposed methodology integrates heterogeneous base learners (GPR, SVM, LSBoost, and ANN) within an attention-enhanced LSTM meta-learner, enabling adaptive weighting of individual models according to prevailing hydraulic conditions. The results demonstrated that the proposed ensemble consistently outperformed all base models and traditional regression benchmarks across multiple performance indicators, achieving an RMSE of 0.0175, a correlation coefficient of 0.9843, and strong agreement indices under 5-fold cross-validation. These improvements were not isolated cases but were stable across folds, confirming the robustness of the proposed framework. The use of an attention-based LSTM as a meta-model represents a methodological advancement over conventional stacking strategies, as it dynamically captures nonlinear interactions among predictors and emphasizes the most informative base models, rather than relying on fixed or heuristic aggregation schemes. From a hydraulic standpoint, the sensitivity analysis provided physically consistent insights into submerged gate behavior. The dominance of the submergence ratio (y₃/y₁) and the relative downstream depth (y₃/w) aligns well with submerged-flow theory, as these parameters govern tailwater control, jet suppression, and effective head reduction beneath radial gates. This physical interpretability strengthens confidence in the model’s predictions and supports its use in operational calibration and discharge estimation tasks. In practical terms, the proposed model is well suited for real-time decision-support applications. Although model training is computationally demanding, it is performed offline, while real-time deployment requires only the trained meta-model and simple algebraic preprocessing of measured variables. This makes integration into existing gate operation and calibration workflows feasible, particularly within SCADA-based irrigation management systems. Nevertheless, the model’s reliability is highest within the range of hydraulic conditions represented in the training data, and caution is required under extreme or extrapolative flow regimes. Despite its strong performance, several limitations should be acknowledged. The model is trained on site-specific field data, and direct transfer to other regulators or gate geometries may require additional calibration or retraining. Measurement noise, missing inputs, and rare extreme operating conditions may also affect prediction reliability. These limitations highlight the importance of continued data collection and model updating in real-world applications. Future research should focus on extending the framework through transfer learning across different gate geometries and sites, incorporating formal uncertainty quantification to support risk-aware operational decisions, and expanding the model to cover transitional free–submerged flow regimes. Overall, this study demonstrates that advanced deep learning–based stacking ensembles can provide accurate, physically consistent, and operationally meaningful tools for discharge coefficient estimation in submerged radial gate systems, offering tangible benefits for modern water resources management. The study recommends future investigations incorporating broader, transfer learning across different gate geometries, Incorporation of uncertainty quantification, Expansion to free–submerged transitional flow regimes.

## Supplementary Information

Below is the link to the electronic supplementary material.

Supplementary Material 1

## Data Availability

The datasets used and/or analysed during the current study available from the corresponding author on reasonable request.
