# Supplementary material for: A novel stacking ensemble model for predicting discharge coefficient of submerged multi parallel radial gates
Source: Sci Rep. 2026 Mar 3;16:7953. doi: 10.1038/s41598-026-38117-2 (PMC12957450; doi:10.1038/s41598-026-38117-2)
Supplement: Supplementary file 1 — Supplementary Material 1 [file 41598_2026_38117_MOESM1_ESM.docx]

- Ensemble model is used.
- Applied to regulators on the Nile.
- Superior performance vs other models.
